# The predictive value of serum level of cystatin C for COVID-19 severity

**DOI:** 10.1038/s41598-021-01570-2

**Published:** 2021-11-09

**Authors:** Luanfeng Lin, Xiaoling Chen, Junnian Chen, Xiaobin Pan, Pincang Xia, Hailong Lin, Houwei Du

**Affiliations:** 1 Department of Infectious Disease, Mengchao Hepatobiliary Hospital of Fujian Medical University, Fuzhou, China; 2 Department of Infectious Disease, Fujian Medical University Union Hospital, Fuzhou, China; 3 Department of Critical Care Medicine, Fujian Medical University Union Hospital, Fuzhou, China; 4 Department of Critical Care Medicine, Fujian Provincial Hospital South Branch, Fuzhou, China; 5 Fujian Center for Disease Control and Prevention, Fuzhou, China; 6 Department of Radiology, Fujian Medical University Union Hospital, Fuzhou, China; 7 Department of Neurology, Fujian Medical University Union Hospital, 29 Xinquan Road, Gulou District, Fuzhou, 350001 China; 8 Institute of Clinical Neurology, Fujian Medical University, Fuzhou, China

**Keywords:** Viral infection, Risk factors

## Abstract

To investigate the potential prognostic value of Serum cystatin C (sCys C) in patients with COVID-19 and determine the association of sCys C with severe COVID-19 illness. We performed a retrospective review of medical records of 162 (61.7 ± 13.5 years) patients with COVID-19. We assessed the predictive accuracy of sCys C for COVID-19 severity by the receiver operating characteristic (ROC) curve analysis. The participants were divided into two groups based on the sCys C cut-off value. We evaluated the association between high sCys C level and the development of severe COVID-19 disease, using a COX proportional hazards regression model. The area under the ROC curve was 0.708 (95% CI 0.594–0.822), the cut-off value was 1.245 (mg/L), and the sensitivity and specificity was 79.1% and 60.7%, respectively. A multivariable Cox analysis showed that a higher level of sCys C (adjusted HR 2.78 95% CI 1.25–6.18, *p* = 0.012) was significantly associated with an increased risk of developing a severe COVID-19 illness. Patients with a higher sCys C level have an increased risk of severe COVID-19 disease. Our findings suggest that early assessing sCys C could help to identify potential severe COVID-19 patients.

## Introduction

In late December 2019, Wuhan, China, a highly infectious respiratory illness due to severe acute respiratory syndrome coronavirus 2 (SARS-CoV-2) infection was first reported. This infectious disease was later designated coronavirus disease 2019 (COVID-19)^1^. As of 8 September 2021, the COVID-19 pandemic has had an unprecedented impact on a global scale with over 220 million cases identified and over four million deaths^2^. Given the uncontrolled global spread of the COVID-19 pandemic, it is essential to find an easily accessible sensitive biomarker for predicting the severe COVID-19 disease.

Accumulating evidence has supported that COVID-19 comprises a systematic endothelial dysfunction^3–5^. In addition to the impaired respiratory function and immune system, the kidney might also be one of the main organs affected^6^. The severity of renal function impairment diverse, ranging from elevated blood urea nitrogen (Bun) or serum creatinine (sCr) levels, to acute kidney injury (AKI) and renal failure^6,7^. A meta-analysis^8^ has shown that the incidence of AKI was more prevalent in severe (2.8% [95% CI 1.4–4.2%]) or critical (36.4% [95% CI 14.6–58.3%]) COVID-19 patients than in mild or moderate cases (1.3% [95% CI 0.2–2.4%]), suggesting an association between renal impairment and COVID-19 severity. Cystatin C is a low molecular mass protein (13.3 KD) produced by most nucleated cells. Blood Cystatin C levels are not influenced by ingestion of meat and no tubular secretion of cystatin, and production of Cystatin C is influenced less by age, gender, and muscle mass^9^. Serum cystatin C (sCys C) is considered a more sensitive biomarker for early renal insufficiency than conventional indicators such as Bun and sCr^10^. To our knowledge, little is known about the prognostic value of sCys C for COVID-19 severity. We hypothesized that in patients infected with SARS-CoV-2 elevated sCys C increases the risk of developing severe illness. We therefore investigated the association between the sCys C and severe COVID-19 disease in this retrospective observational study.

## Methods

### Ethics approval and consent to participate

The ethics committee of Fujian Medical University Union Hospital approved the study protocol (NO.2020GFKY005). All clinical investigations were conducted based on the principles expressed in the declaration of Helsinki. Written informed consent was waived by the ethical committee due to the retrospective nature of our study of routine clinical data.

### Study design and participants

This is a single-center, retrospective, observational study done at Tumor Center of Union Hospital, Tongji Medical College, Huazhong University of Science and Technology (Wuhan, China), a designated hospital to treat patients with COVID-19. We analyzed consecutive patients admitted between 15 February and 14 March 2020 because of a COVID-19 disease based on World Health Organization interim guidance^11^. Laboratory confirmation of COVID-19 infection was performed using Reverse Transcription-Polymerase Chain Reaction (RT-PCR) detection by the local health authority as previously described^12^.

### Data collection and outcome measures

We retrospectively reviewed the electronic medical records of 168 consecutive eligible patients with COVID-19 using a digital database. We extracted the epidemiological, demographic, clinical, laboratory data on admission, the chest computed tomography (CT) image, and outcome data using a standardized data collection form. In cases of disagreement, we reached a consensus after team discussion. In case of missing or uncertain data, we obtained and clarified data by direct consulting with attending doctors and other healthcare providers. We constructed a vascular risk factor score based on the following well-documented vascular risk factors in each patient: hypertension, diabetes, dyslipidemia, atrial fibrillation, current smoker, overweight and physical inactivity^13–17^. Quick Sequential Organ Failure Assessment (qSOFA) was calculated based on data regarding systolic blood pressure, respiratory rate, and mental status within 24 h after admission^18^ Our primary outcome was severe COVID-19 disease defined as fever or suspected respiratory infection, plus one of: respiratory rate > 30 breaths/min; severe respiratory distress; or SPO_2_ ≤ 93% on room air based on the interim guidance of the World Health Organization^11^. Our secondary outcome was death, which was limited by the duration of our observation period. All the authors agreed on the study protocol and reviewed the manuscript.

### Measurements of cystatin C, creatinine and Bun

Serum cystatin C was measured by a turbidimetric immunoinhibition assay using the cystatin kit (Tina-quant cystatin C Gen. 2, Roche) with an automatic biochemical analyzer (Cobas c-system, Roche, Switzerland). This method is traceable to a primary reference material with values assigned by the international cystatin C reference material (ERM-DA471/IFCC) as a calibrator^19^. This procedure had a total coefficient of variation of 2.2% at a cystatin C level of 1.0 mg/L and of 1.4% at a level of 4.0 mg/L. Reference range 0.55–1.09 mg/L. Serum creatinine was determined by a traceable method (enzymatic assay calibrated against the National Institute of Standards and Technology standard reference material [SRM 967]) with an automatic biochemical analyzer (Cobas c-system, Roche, Switzerland)^20^. The total coefficient of variation is < 4.0%, and the reference range is 44–133 μmol/L. Serum Bun was determined by an enzymatic method with an automatic biochemical analyzer (Cobas c-system, Roche, Switzerland) according to the kit protocol. This method is traceable to a primary reference material (SRM 909b). This procedure had a total coefficient of variation of 1.2% at a level of 7.2 mmol/L and of 0.7% at a level of 35.1 mmol/L. The reference range is 2.9–8.2 mmol/L.

### Statistical analysis

We summarized continuous and categorical variables as median (interquartile range, IQR) and numbers (percentages), respectively. We used the t-test or Mann–Whitney test to compare differences in continuous variables, and the chi-square test or Fisher's exact test to compare differences in categorical variables where appropriate. The classification performance of sCys C, Bun and sCr to discriminate between severe and non-severe cases was evaluated by calculating the area under the ROC curve (AUROC) and its 95% confidence intervals (95% CI). We defined the score with the largest Youden Index as the optimal cut-off value for predicting COVID-19 severity. Based on the optimal cutoff value, we calculated the sensitivity and specificity. The differences between AUROC were evaluated by using a method as previously described^21^. We divided patients into higher and lower group based on sCysC levels above and below the cut­off value. We calculated the absolute event rate per 1000 patient-days for severe COVID-19 illness and death during our observation period. Kaplan–Meier curves depicted the risk for outcome events stratified by sCys C levels (higher vs lower). We implemented univariable and multivariable COX proportional hazards regression models to examine the association between sCys C level and the risk of developing a severe COVID-19. For multivariate analysis, to avoid overfitting in the model, we chose the variables based on previous findings and clinical constraints. Previous studies have shown older age, qSOFA, vascular risk factor burden, and several laboratory abnormal findings to be associated with an increased risk of severe COVID-19^13,18^. Therefore, we chose qSOFA, vascular risk factor burden, decreased lymphocyte, and increased lactate dehydrogenase (LDH) in addition to age and sex for our multivariable regression model. All analyses were performed using STATA 12.0 (StataCorp LP, College Station, TX) and SPSS for Windows (SPSS 25.0, IBM, Inc., Chicago, IL, USA).

## Results

### Baseline difference between patients with severe and non-severe COVID-19

**Figure 1:**
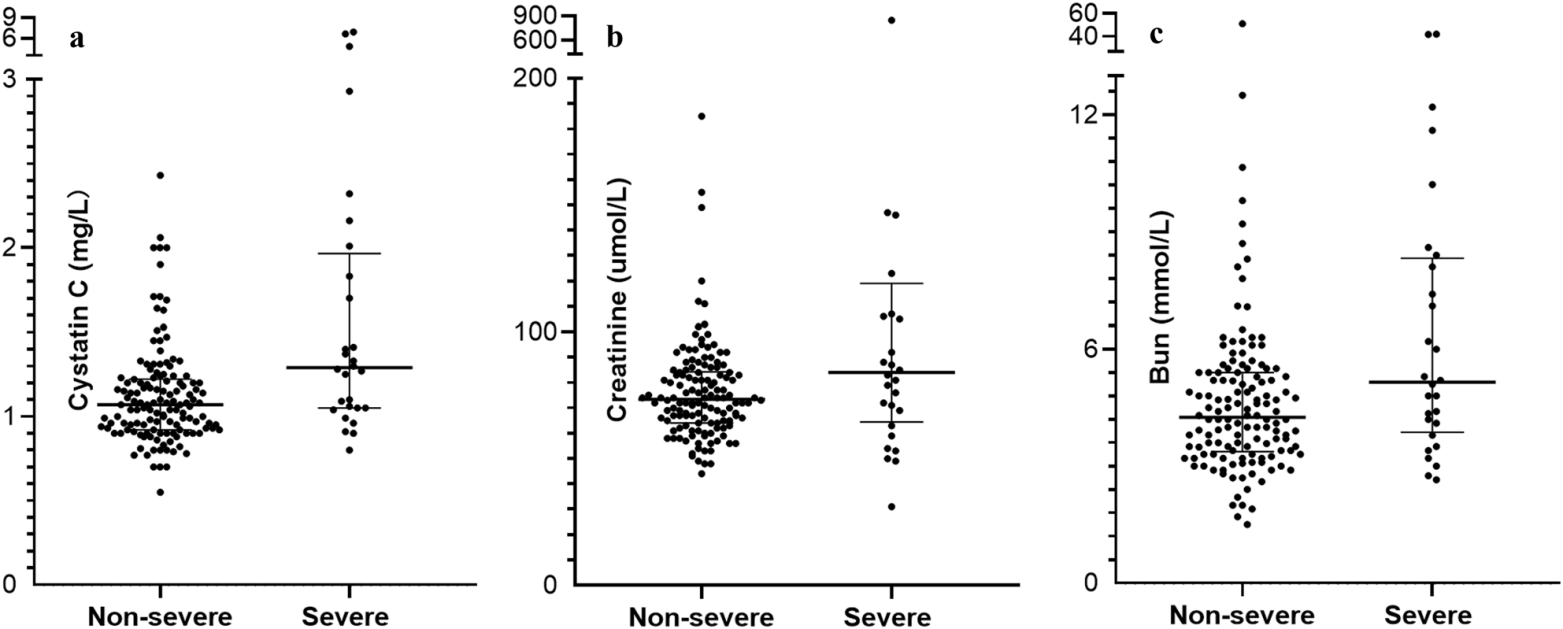
Compariosn of serum levels of cystatin C, creatinine, and Bun in COVID-19 patients. (**a**) Serum level of cystatin C in severe and non-severe COVID-19 patients; (**b**) Serum level of creatinine in severe and non-severe COVID-19 patients; (**c**) Serum level of Bun in severe and non-severe COVID-19 patients; Abbreviations: COVID-19 = coronavirus disease 2019, Bun = blood urea nitrogen, sCr = serum creatinine.

We admitted 168 consecutive patients with COVID-19 from 15 February through 14 March 2020. After excluded four suspected patients and two cases with incomplete data, we included 162 laboratory-confirmed COVID-19 patients (61.7 ± 13.5 years) in the final analysis. Table 1 summarized the demographics, clinical and radiological characteristics in patients with severe COVID-19 illness and non-severe COVID-19 illness. Patients with severe COVID-19 disease were not significantly different from those with non-severe illness regarding their most previous history, exposure to wet seafood market, and onset symptoms. Patients with severe disease were older (70.1 ± 12.7 vs 59.8 ± 13.0, *p* < 0.001), more likely to be male (20 [71.4%] vs 64 [47.8%], *p* = 0.023), and more likely to have pre-existent cardio-cerebrovascular disease (8 [28.6%] vs 15 [11.2%], *p* = 0.017). Regarding routine blood test findings, patients with severe COVID-19 disease were more likely to have decreased lymphocytes (64.3% vs 26.1%, *p* < 0.001) and increased LDH level (60.7% vs 24.6%, *p* < 0.001). Supplementary Figs. 1a, 1b, and 1c show the distribution of sCys C, creatinine and Bun levels in severe and non-severe COVID-19 patients. sCys C was significantly increased in patients with severe COVID-19 compared to non-severe patients (1.28 [1.05–1.83] mg/L vs 1.07 [0.92–1.22], *p* = 0.001, Fig. 1a). Figure 1b,c show the serum levels of creatinine and Bun were higher in severe than in non-severe COVID-19 patients. Regarding chest CT findings, patients with severe COVID-19 illness were more likely to be bilaterally affected (*p* = 0.024).

**Table 1:** Characteristics at baseline among severe and non-severe COVID-19 patients.

	Total (n = 162)	Severe (n = 28)	Non-severe (n = 134)	*P*-value
Age, (y) mean ± SD	61.7 ± 13.5	70.1 ± 12.7	59.8 ± 13.0	< 0.001
Male, n (%)	84 (51.9)	20 (71.4)	64 (47.8)	0.023
Current smoker, n (%)	17 (10.5)	2 (7.1)	15 (11.2)	0.766
Often drinker, n (%)	3 (1.9)	0	3 (2.2)	> 0.999
Hypertension, n (%)	50 (30.9)	12 (42.9)	38 (28.4)	0.131
Diabetes, n (%)	30 (18.5)	7(25.0)	23 (17.2)	0.332
COPD, n (%)	12 (7.4)	4 (13.4)	8 (6)	0.258
Cardio-cerebrovascular disease, n (%)	23 (14.2)	8 (28.6)	15 (11.2)	0.017
Digestive disease, n (%)	15 (9.3)	1 (3.6)	14 (10.4)	0.433
Previous tumor, n (%)	13 (8.0)	3 (10.7)	10 (7.5)	0.847
Immunosuppresive, n (%)	3 (1.9)	1 (1.6)	2 (1.5)	> 0.999
Wet market exposure, n (%)	2 (1.2)	1 (3.6)	1 (0.7)	0.772
**Clinical manifestion**				
Fever, n (%)	114 (70.4)	19 (67.9)	95 (70.9)	0.749
Dry cough, n, (%)	103 (63.6)	19 (67.9)	84 (62.7)	0.605
Productive cough, n (%)	23 (14.2)	2 (7.1)	21 (15.7)	0.38
Fatigue, n (%)	56 (34.6)	9 (32.1)	47 (35.1)	0.767
Musle or joint ache, n (%)	21(13.0)	2(7.1)	19 (14.2)	0.485
Thoracalgia, n (%)	31(19.1)	6(21.4)	25(18.7)	0.735
Sore throat, n (%)	23 (14.2)	4 (14.3)	19 (14.2)	> 0.999
Diarrhea, n (%)	12 (7.4)	3 (10.7)	9 (6.7)	0.735
Catarrh, n (%)	6 (3.7)	0	6 (4.5)	0.591
Anorexia, n (%)	47 (29.0)	28 (28.6)	39 (29.1)	0.955
Short of breath, n (%)	65 (40.1)	15 (53.6)	50 (37.3)	0.11
Headache, n (%)	19 (11.7)	3 (10.7)	16 (11.9)	> 0.999
Total symptoms, (IQR)	3 [2–4]	3 [2–4]	3 [2–4]	0.815
**Regular blood test**				
WBC × 10^9^/L (IQR)	5.4 [4.4–7.3]	5.2 [3.9–10.0]	5.4 [4.4–7.1]	0.643
Decreased WBC, n (%)	10 (6.2)	4 (14.3)	6 (4.5)	0.126
Lymphocytes × 10^9^/L (IQR)	1.4 [0.9–1.7]	0.8 [0.6–1.4]	1.4 [1.1–1.7]	< 0.001
Decreased lymphocytes, n (%)	53 (32.7)	18 (64.3)	35 (26.1)	0.001
sCys C (mg/L)	1.08 [0.94–1.28]	1.28 [1.05–1.83]	1.07 [0.92–1.22]	0.001
Bun (mmol/L)	4.4 [3.4–5.5]	5.1 [3.8–8.4]	4.3 [3.9–5.4]	0.014
sCr (umol/L)	74 [64–87]	83 [63–107]	74 [64–84]	0.042
LDH (U/L)	197 [164–264]	271 [203–338]	187 [160–244]	< 0. 001
Increased LDH, n (%)	50 [30.9]	17 [60.7]	33 [24.6]	< 0. 001
**CT findings, n (%)**				0.024
Unilateral pneumonia, n (%)	25 (15.4)	2 (7.1)	23 (17.2)	
Bilateral pneumonia, n (%)	85(52.5)	11 (39.3)	74 (55.2)	
Multiple mottling and Ground-glass opacity, n (%)	52 (32.1)	15 (53.6)	37 (27.6)	

Legend and abbreviations: COVID-19 = coronavirus disease 2019; SD = Standard deviation; COPD = Chronic obstructive pulmonary disease; IQR = Interquartile range; WBC = white blood cell (10^9^/L; reference range 3.5–9.5); Lymphocytes (× 10^9^/L; reference range 1.1–3.2); sCys C = serum cystatin C (mg/L, reference range 0.55–1.09); Bun = blood urea nitrogen (mmol/L; reference range 2.9–8.2), sCr = serum creatinine (μmol/L; reference range 44–133), LDH = Lactate dehydrogenase (U/L; reference range 109–245); CT = Computed tomography

### Receiver operating characteristic curve analysis

**Figure 2:**
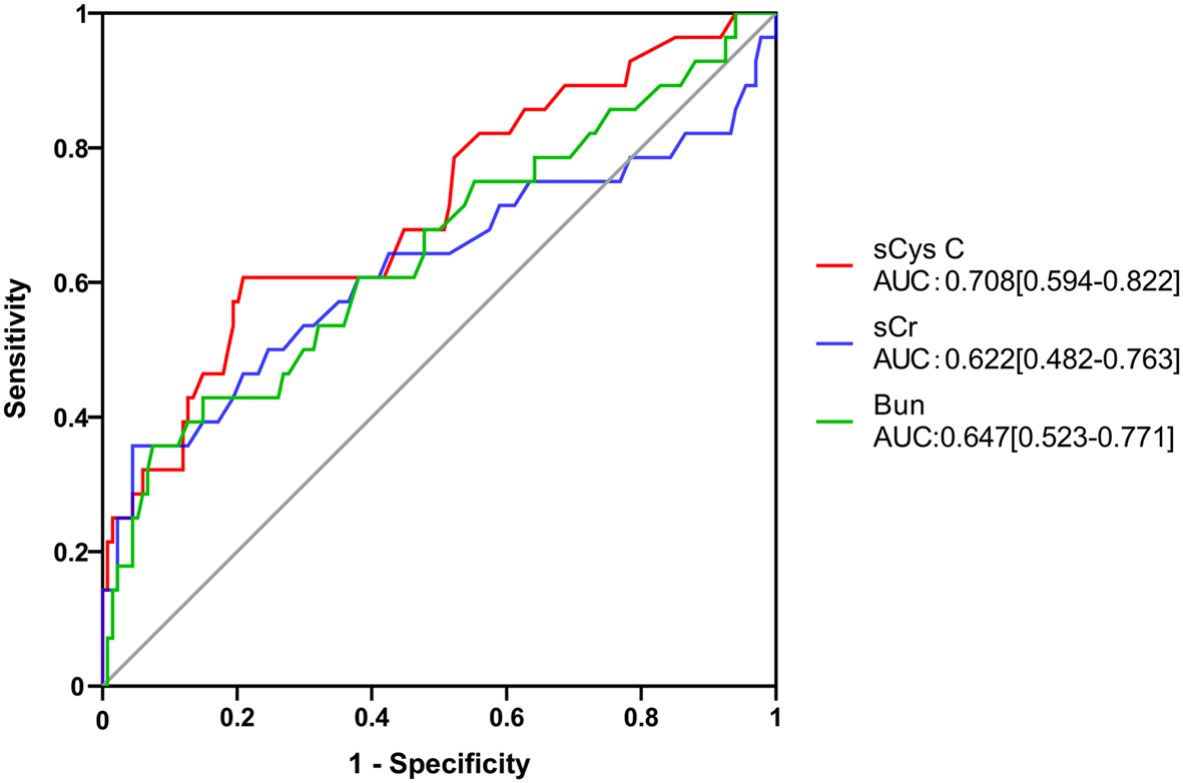
Predictive performances of sCys C, Bun, and sCr using ROC analysis. Comparison of predictive performances of sCys C, Bun, and sCr. Abbreviations: COVID-19 = coronavirus disease 2019, sCys C = serum cystatin C, Bun = blood urea nitrogen, sCr = serum creatinine, ROC = receiver operating characteristic curve.

The ROC curves of sCys C value for predicting severe COVID-19 illness was shown in Fig. 2. sCys C resulted numerically in a higher AUROC 0.708 (95% CI 0.594–0.822) than Bun and sCr; 0.622 (95% CI 0.482–0.763) and 0.647 (95% CI 0.523–0.771), respectively (Table 2). The difference was statistically different (*p* = 0.037). Based on the largest Youden Index (0.398), an optimum cut-off value of 1.245 (mg/L) was used to predict severe COVID-19 illness by using the sCys C, with a sensitivity and specificity of 79.1% and 60.7%, respectively.

**Table 2:** Predictive performance of sCys C, Bun and sCr for severe COVID-19.

	AUC	95% CI	*P* value
sCys C	0.708	0.594–0.822	0.037*
Bun	0.622	0.482–0.763	
sCr	0.647	0.523–0.771	

Abbreviations: sCys C = serum cystatin C; Bun = blood urea nitrogen, sCr = serum creatinine, COVID-19 = coronavirus disease 2019, AUROC = Area under the receiver operating characteristic curve;

*P = 0.037 (Comparison of AUROC of sCys C, Bun and sCr).

### Survival analysis and COX proportional hazards regression analysis

**Figure 3:**
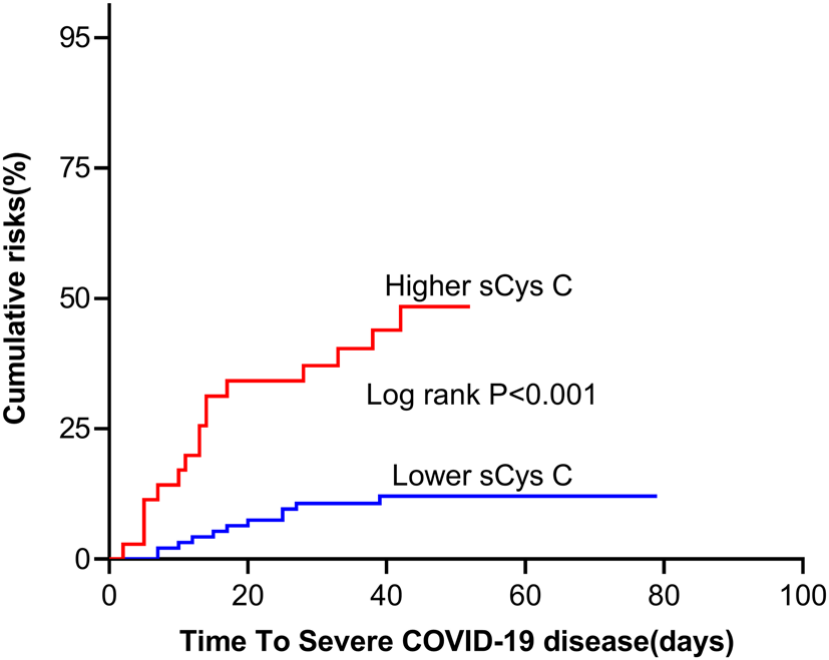
Cumulative probability of severe COVID-19 disease stratified by sCys C level. Higher level means > 1.245 (mg/L); Lower level level means < 1.245 (mg/L). Abbreviations: COVID-19 = coronavirus disease 2019; sCys C = serum cystatin C.

The median follow-up time was 42 days [IQR 35–49], providing 6430 patient-days of data. Twenty-eight patients (17.3%) developed severe COVID-19 illness with a median (14 [7–25] days) duration between symptom onset to developing a severe disease, an event rate of 4.35 per 1000-patient days (95% CI 3.01–6.31). Six patients (3.7%) died during hospitalization, an event rate of 0.85 per 1000-patient days (95% CI 0.38–1.90). Kaplan–Meier curves showed an increase in the risk of severe COVID-19 illness (log rank *p* < 0.001, Fig. 3). In univariable analysis, a higher sCys C level was associated with an increased risk of severe COVID-19 (unadjusted HR 4.95, 95% CI 2.31–10.57). After adjustment for age and sex, a higher sCys C level remained significantly associated with severe COVID-19 (adjusted HR 3.04 95% CI 1.30–7.13). This relationship did not change after additional adjustment for decreased lymphocyte, increased LDH, qSOFA or vascular risk factor burden along with age and sex (Table 3). A sensitivity analysis excluding three patients who reported previous renal disease resulted in similar findings (Table 4).

**Table 3:** Association between higher sCys C level and severe COVID-19 illness.

Unadjusted		Adjusted for age and sex		Multivariable-adjusted mode l		Multivariable-adjusted model 2	
HR (95% CI)	*P* value	HR (95% CI)	*P * value	HR (95% CI)	*P* value	HR (95% CI)	*P *value
4.95 [2.31–10.57]	< 0.001	3.04 [1.30–7.13]	0.01	2.78 [1.25–6.18]	0.012	2.75 [1.23–6.14]	0.014

Abbreviations: sCys C = serum cystatin C; COVID-19 = coronavirus disease 2019; HR = Hazards ratio;

Multivariable Model 1: Adjusted for age, sex, increased lactate dehydrogenase, decreased lymphocyte, and qSOFA (Quick Sequential Organ Failure Assessment).

Multivariable Model 2: Adjusted for age, sex, increased lactate dehydrogenase, decreased lymphocyte, and vascular risk factor burden.

**Table 4:** Association between higher sCys C level and severe COVID-19 illness in a sensitivity analysis.

Unadjusted		Adjusted for age and sex		Multivariable-adjusted mode l		Multivariable-adjusted model 2	
HR (95% CI)	*P *value	HR (95% CI)	*P *value	HR (95% CI)	*P *value	HR (95% CI)	*P *value
4.80 [2.30–10.38]	< 0.001	3.07 [1.30–7.27]	0.01	2.80 [1.24–6.31]	0.013	2.70 [1.20–6.05]	0.016

Abbreviations: sCys C = serum cystatin C; COVID-19 = coronavirus disease 2019; HR = Hazards ratio;

Multivariable Model 1: Adjusted for age, sex, increased lactate dehydrogenase, decreased lymphocyte, and qSOFA (Quick Sequential Organ Failure Assessment).

Multivariable Model 2: Adjusted for age, sex, increased lactate dehydrogenase, decreased lymphocyte, and vascular risk factor burden.

## Discussion

The early and fast evaluation of severely ill COVID-19 patients is paramount to ensure early medical monitoring and interventions for these patients due to the medical resource constraints during the COVID-19 pandemic. Our most important finding is that in COVID-19 patients a higher sCys C level is associated with an increased risk of experiencing severe COVID-19 illness. Our results highlighted the considerable predictive performance of sCys C for severe COVID-19 disease on ROC curve analysis. At the time of admission, a sCys C value over 1.245 (mg/L) was highly predictive of developing a severe COVID-19 disease. Our findings were in line with a previous hospital-based study that showed the highest baseline sCys C level was associated with more severe inflammatory status and unfavorable outcomes among COVID-19 patients^22^. These findings suggest that sCys C could serve as a potential inflammatory target for preventing COVID-19 from the likely progression of critical illness and mortality, in addition to representing early renal insufficiency. Since the sCys C is generally readily available at hospital admission, our findings may contribute to identify COVID-19 patients with poor prognosis at an early stage.

Our ROC analysis showed that sCys C had a better predictive performance for severe COVID-19 than two conventional renal function indicators (Bun and sCr). Accumulating evidence has demonstrated that Bun and sCr levels might not be good indicators for the early detection of renal injury^23,24^. sCys C is emerging as a novel index with a high sensitivity and specificity for evaluating renal function, independently of age, sex, weight, inflammation, and other factors^25,26^. The renal clearance of 5–40 kDa molecules like cystatin C (13.3 KDa) is decreased more than that of low molecules like creatinine (0.133 kDa)^27^. Since cystatin C is mainly excreted via glomerular transport, a reduction in their glomerular filtration rate would result in a simultaneous increase of sCys C levels. Previous studies suggest that a decrease in the pore diameters of the functional pores might account for the increased sCys C levels^28,29^. Recently, researchers from Lund University (Sweden) proposed the designation ‘Shrunken pore syndrome’ to reflect a reduced pore size of the glomerular membranes, which might help explain the superiority of cystatin C as a predictor of mortality^30,31^. In line with a previous study^32^, our findings that sCys C was significantly increased in patients with severe COVID-19 compared to those with non-severe COVID-19 illness (1.28 [1.05–1.83] vs 1.07 [0.92–1.22]) indicate that severe SARS-CoV-2 infection might damage the kidney. Our findings were supported by a previous hospital-based observational study that showed COVID-19 patients with renal involvement had a mortality of 11.2%, compared to 1.2% in those without renal involvement^33^. These results suggest that renal complications in COVID-19 were associated with higher mortality.

Our findings have important implications for early COVID-19 prevention and treatment. For example, hydroxychloroquine is a safe and highly tolerable approach with minimum side effects. A retrospective longitudinal cohort study showed that hydroxychloroquine treatment (OR 3.891, 95% CI 1.196–12.653) was associated with renal function recovery in patients with lupus nephritis^34^. A large-sampled multicentered observational study has shown that the exposure of hydroxychloroquine is associated with a decreased risk of hospitalization from COVID-19 (OR 0.53; 95% CI 0.29–0.95)^35^. Additional, findings from some recent random-controlled studies raise the concern about whether dexamethasone treatment could improve the outcome of COVID-19^36,37^ Whether patients with higher sCys C levels might benefit form early hospital treatment measures such as hydroxychloroquine or dexamethasone need to be addressed in future large-scale studies.

The underlying mechanisms of the association between sCys C level and COVID-19 severity remain unclear. SARS-CoV-2 infection may directly cause endothelial damage, infects renal tubular epithelium and podocytes, causing mitochondrial dysfunction and acute tubular necrosis. This process is mediated through the angiotensin-converting-enzyme 2 (ACE2) dependent pathway^38,39^ Moreover, available evidence suopport that kidney impairment in COVID-19 patients may be caused by an interplay of virus-mediated injury, a dysregulated inflammatory response, hypercoagulation, and microangiopathy^40^. A severe inflammatory process during SARS-CoV-2 infection might also probably induce increased levels of cystatin C similar in size to cytokines like interleukin-6. This hypothesis is supported by a previous study showing that sCys C level was positively correlated with inflammatory indicators such as IL-6, tumor necrosis factor-α, and hypersensitive C-reactive protein^41^. These aforementioned inflammatory indicators have been reported to be related to the COVID-19 severity^42,43^. The underlying interaction between circulating cystatin C and viral infection may provide insight into our better understanding of pathophysiological events in COVID-19.

The results of the present study should be interpreted within the constraints of its limitations. First, we conducted this retrospective study at a single-centered hospital with limited sample size. Moreover, we retrospectively collected data from medical records and laboratory data regarding prior renal function in some patients was not accessible. Therefore, we may underestimate the true overall incidence of COVID-19-associated elevated sCys C. However, a sensitivity analysis excluding those patients who reported previous renal disease did not alter the association between a higher sCys C level and a higher COVID-19 risk. Notably, we did not include data regarding urine output, a defining characteristic of acute kidney insufficiency. Therefore, more studies with a broad geographic scope are needed to get a more comprehensive understanding of role of renal impairment in COVID-19. Our strength include the association of sCys C with the COVID-19 severity was verified using previous well-validated cofounders in consecutive laboratory-confirmed participants.

## Conclusions

Our findings that COVID-19 patients with a higher sCys C level were at an increased risk suggest that this population needs early prevention and treatment.

## Supplementary Information

Supplementary Information.

## Data Availability

The datasets used and/or analyzed during the current study are available from the corresponding author on reasonable request (Email: houweidu@fjmu.edu.cn).
