# Supplementary material for: The predictive value of serum level of cystatin C for COVID-19 severity
Source: Sci Rep. 2021 Nov 9;11:21964. doi: 10.1038/s41598-021-01570-2 (PMC8578213; doi:10.1038/s41598-021-01570-2)
Supplement: Supplementary file 1 — Supplementary Information. [file 41598_2021_1570_MOESM1_ESM.pdf]

Title: The predictive value of serum level of cystatin C for COVID-19 severity

Luanfeng Lin, M.D., Xiaoling Chen, M.D., Junnian Chen, M.D., Xiaobin Pan, M.D.[4], Pincang Xia, M.D. [5], Hailong Lin, M.D. [6], Houwei Du, M.D. [7,8*]

Supplementary materials

Supplementary figure 1: Distribution of the sCys C, Creatinine, and Bun values in severe and non-severe COVID-19 patients

Fig S1a

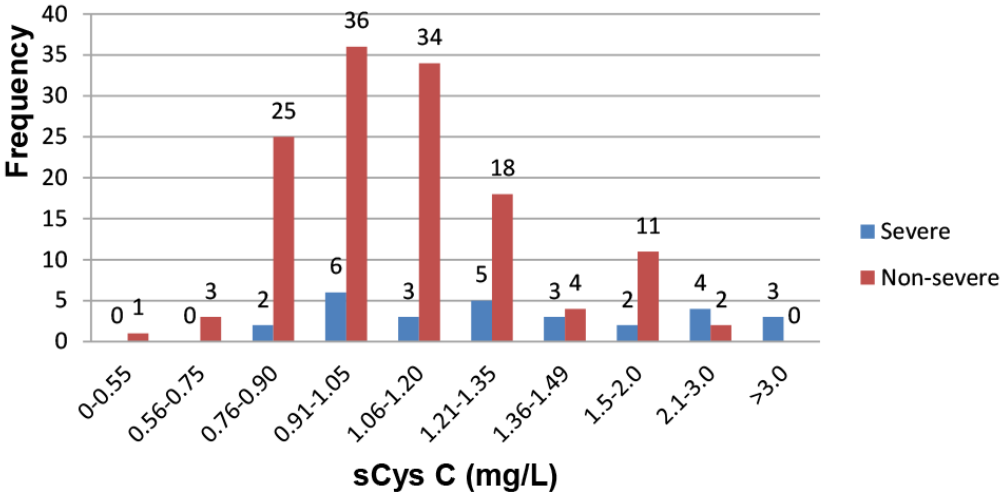

Fig S1b

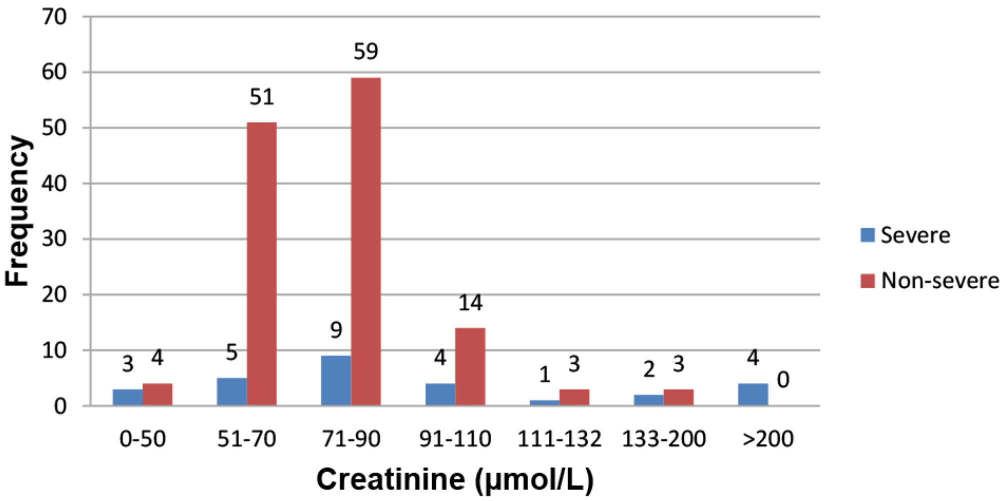

Fig S1c

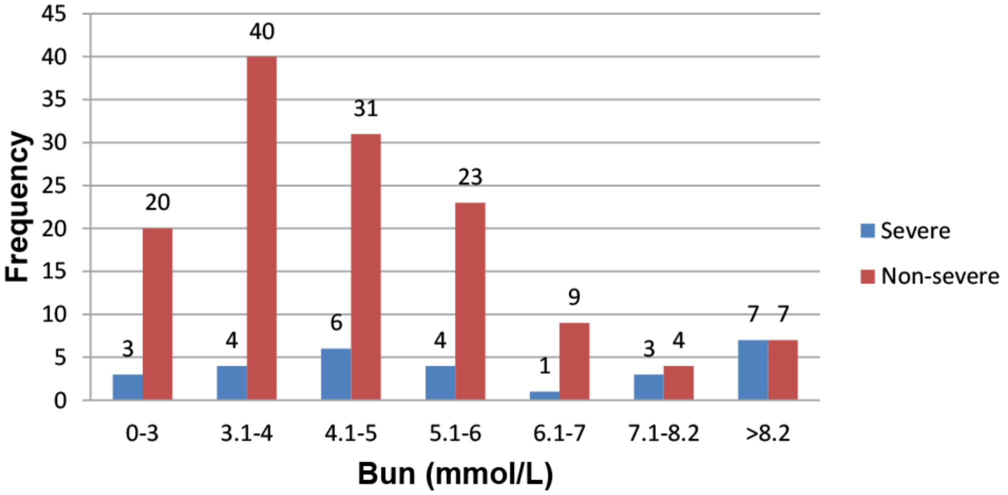

Supplementary figure 1: Distribution of the sCys C, Creatinine, and Bun values in severe and non-severe COVID-19 patients

Figure legend: 1a: Distribution of the sCys C levels in severe and non-severe COVID-19 patients; 1b: Distribution of serum creatinine levels in severe and non-severe COVID-19 patients; 1c: Distribution of serum Bun levels in severe and non-severe COVID-19 patients

Abbreviations: COVID-19 = coronavirus disease 2019, sCys C = serum cystatin C, Bun = blood urea nitrogen
